# Texas Quarantine

**Published:** 1886-04

**Authors:** 


### QUARANTINE IN TEXAS.

The Governor of Texas (head of health department) has issued his proclamation instituting quarantine against all infected ports from and after May 1st, prox. This is done every year.

